# Linguistic validation of the simplified Chinese version of the US National Cancer Institute’s patient-reported outcomes version of the common terminology criteria for adverse events (PRO-CTCAE™)

**DOI:** 10.1186/s12885-020-07631-5

**Published:** 2020-11-26

**Authors:** Cheng KKF, S. A. Mitchell, N. Chan, E. Ang, W. Tam, R. Kanesvaran

**Affiliations:** 1 Alice Lee Centre for Nursing Studies, Yong Loo Lin School of Medicine, National University of Singapore, Singapore, Singapore; 2 Division of Cancer Control and Population Sciences, National Cancer Institute, Rockville, USA; 3 Division of Palliative Care, National University Cancer Institute, Singapore, Singapore; 4 Division of Medical Oncology, National Cancer Centre Singapore, Singapore, Singapore

**Keywords:** PRO-CTCAE, Patient-reported outcomes, Symptomatic adverse events, Linguistic validation

## Abstract

**Background:**

The aim of this study was to translate and linguistically validate the U.S. National Cancer Institute’s Patient-Reported Outcomes version of the Common Terminology Criteria for Adverse Events (PRO-CTCAE™) into Simplified Chinese for use in Singapore.

**Methods:**

All 124 items of the English source PRO-CTCAE item library were translated into Simplified Chinese using internationally established translation procedures. Two rounds of cognitive interviews were conducted with 96 cancer patients undergoing adjuvant treatment to determine if the translations adequately captured the PRO-CTCAE source concepts, and to evaluate comprehension, clarity and ease of judgement. Interview probes addressed the 78 PRO-CTCAE symptom terms (e.g. fatigue), as well as the attributes (e.g. severity), response choices, and phrasing of ‘at its worst’. Items that met the a priori threshold of ≥20% of participants with comprehension difficulties were considered for rephrasing and retesting. Items where < 20% of the sample experienced comprehension difficulties were also considered for rephrasing if better phrasing options were available.

**Results:**

A majority of PRO-CTCAE-Simplified Chinese items were well comprehended by participants in Round 1. One item posed difficulties in ≥20% and was revised. Two items presented difficulties in < 20% but were revised as there were preferred alternative phrasings. Twenty-four items presented difficulties in < 10% of respondents. Of these, eleven items were revised to an alternative preferred phrasing, four items were revised to include synonyms. Revised items were tested in Round 2 and demonstrated satisfactory comprehension.

**Conclusions:**

PRO-CTCAE-Simplified Chinese has been successfully developed and linguistically validated in a sample of cancer patients residing in Singapore.

## Background

There is increasing recognition in both clinical research and in care delivery of the importance of using patient-reported outcomes (PROs) to directly capture the patient’s perspective of their treatment experiences, including treatment-related toxicities. A recent review highlighted that the measurement of PROs could provide considerable added value towards the interpretation of study-related outcomes by both trialists and clinicians, and may also strengthen the quality of clinician-patient interactions, and assist regulatory agencies with decision-making [1].

The Patient-Reported Version of the Common Terminology Criteria for Adverse Events (PRO-CTCAE™), was developed by the US National Cancer Institute (NCI), to be used in conjunction with the Common Terminology Criteria for Adverse Events (CTCAE) to facilitate patient self-reporting of symptomatic adverse effects. PRO-CTCAE is designed to supplement clinician-based CTCAE grading, leading to improved characterization and reporting of cancer treatment toxicities [2–4]. CTCAE is the standard for grading and reporting toxicities in cancer clinical trials, and is used to guide treatment decision-making and inform the labelling of oncology products [4, 5]. The most recent version of the clinician-based CTCAE, version 5, is a compendium of 790 discrete adverse events each of which is defined and graded using an ordinal severity scale [3]. Of the 790 adverse events listed in the CTCAE, 78 symptomatic adverse events that are amenable to patient self-reporting (e.g. pain, fatigue) were selected for inclusion in PRO-CTCAE [2, 6].

The PRO-CTCAE item library is comprised of 124 items reflecting 78 symptomatic toxicities. PRO-CTCAE items evaluate these symptomatic toxicities with respect to the attributes of frequency, severity, interference with usual or daily activities, presence/absence or amount. PRO-CTCAE items direct the respondent to report the symptomatic adverse event at its worst during the past 7 days. Each PRO-CTCAE item includes a plain language term describing the symptomatic adverse event, and one or more of the attributes that align with the CTCAE grading criteria for that adverse event [2–4]. Iterative cognitive debriefing interviews conducted with 127 participants with a diverse range of cancer diagnoses and lower levels of educational attainment provide evidence that PRO-CTCAE, including the terms, attributes, response options, and recall period, is well understood and meaningful to patients receiving treatment for cancer, thus supporting the content validity of this instrument [4]. A previous validation analysis of the PRO-CTCAE involving 940 participants undergoing cancer treatment demonstrated favourable test-retest reliability, construct validity, and responsiveness [3]. The original English PRO-CTCAE has been translated and linguistically validated in more than 20 languages [6–9]. There is a need for development of a Simplified Chinese-language version of PRO-CTCAE to make the PRO-CTCAE item library accessible to a larger number of patients with cancer. This is important since Chinese-speaking populations constitute approximately 16% of the global population [10]. Quality translation of the original language, and linguistically validation to ensure conceptual equivalence with the original English source and cultural appropriateness, comprehensibility, clarity and ease of response of the translated PRO-CTCAE-Simplified Chinese items are particularly important. Availability of such a linguistically validated Simplified Chinese version of PRO-CTCAE facilitates continued adoption of this measure in cancer clinical trials. In addition, availability of a Simplified Chinese version of PRO-CTCAE would support international cancer research efforts. It would also increase the availability of internationally comparable data of toxicity information from patients receiving cancer treatments, so as to help identify effective and tolerable cancer treatment regimens and guide provision of targeted supportive care to improve tolerability. Therefore, the aim of this study was to develop PRO-CTCAE in the Simplified Chinese language, and to linguistically validate the translation to ensure conceptual equivalence with the PRO-CTCAE English source, and confirm a high level of comprehension, clarity, and ease of response.

## Methods

PRO-CTCAE-Simplified Chinese was developed, tested and refined using internationally established translation procedures proposed by Wild et al. (2005) [11] and the World Health Organization (2016) [12]. The process describes two stages (1): forward and backward translation and (2) linguistic validation using cognitive debriefing interviews.

### Forward and backward translation

Translation procedures were conducted by two translators. One was a bilingual Singapore-residing speaker who was fluent in Mandarin. The other translator was a trilingual Hong Kong Special Administrative Region China-residing linguist. Both translators were well-versed in Mandarin/ written Chinese, Cantonese and English, and had substantial prior experience translating between *English* and Simplified *Chinese in health care contexts. Each translator* performed independent forward translations of the original English language version of PRO-CTCAE into Simplified Chinese. Our goal in selecting translators residing in different Chinese speaking countries was to ensure that item phrasing be well-comprehended and culturally relevant to geographically diverse Chinese-speaking populations. The forward translations were then reconciled and harmonized into one Simplified Chinese version by a bilingual investigator in Singapore and a native Chinese-speaking healthcare professional from China. Next, a bilingual healthcare professional and a second bilingual person from Singapore who had not seen the original English version independently back-translated the reconciled Simplified Chinese translation into English. The backward translations were compared with the original English PRO-CTCAE to identify discrepancies. The initial reconciled Simplified Chinese translation was then further refined in an iterative fashion by two bilingual investigators in Singapore, a native Chinese-speaking healthcare professional from China, and a native Chinese-speaking representative from the US NCI. This resulted in a Simplified Chinese translation of PRO-CTCAE with semantic and conceptual equivalence to the original English PRO-CTCAE and strong suitability for use with Chinese-speakers in diverse regions. This translation was then advanced for linguistic validation in a sample of patients undergoing cancer treatment.

### Cognitive debriefing interviews: materials and methods

The PRO-CTCAE-Simplified Chinese was evaluated through a series of cognitive debriefing interviews with patients undergoing adjuvant treatment. The debriefing interviews were semi-structured and included questions focused on comprehensibility, clarity, ease of judgment, and ease of response. This process aimed to ensure the cultural appropriateness, comprehensibility, clarity and ease of response of the translated PRO-CTCAE-Simplified Chinese items including the symptom terms, item stems, recall period, attributes (i.e. frequency, severity, and interference with usual or daily activities), and response options [4, 13–16]. The study was conducted at two national cancer centers in Singapore following approval from the respective Institutional Review Boards. Individuals were invited to participate if they were native Chinese speakers who were able to read Simplified Chinese, age 21 years or older, and newly diagnosed with colorectal or breast cancer and undergoing adjuvant chemotherapy. We purposively sampled participants with two common cancers, colorectal or breast cancer, to optimize the pace of study accrual and to assure a balanced representation of women and men. In Singapore, colorectal cancer was the most common cancer diagnosed in men, while breast and colorectal cancers are the two most common cancers in women [17]. Clinical staff at study sites screened potential participants. Eligible participants who expressed an interest in participation were referred by their clinician to research assistants who provided a detailed explanation of the study purpose and data collection procedures, and obtained their consent. This study was conducted in accordance with the Declaration of Helsinki; all participants provided written informed consent at enrollment.

We planned for at least two rounds of interviews in this validation study. We used a process of iterative refinement of items and continued cognitive interviewing until all the PRO-CTCAE-Simplified Chinese items demonstrated good comprehension [6]. Two native Chinese-speaking, bilingual trained interviewers from China and Singapore conducted the cognitive debriefing interviews. In Singapore, more than 70% of the residential population is ethnically Chinese. Although English is the official language in Singapore, Mandarin Chinese is commonly spoken in daily life among ethnic Chinese populations, and Simplified Chinese characters are the standard character set of the Chinese written language. Mandarin Chinese and Simplified Chinese characters are also the most widely spoken and written languages, both in China and in other Chinese-speaking communities around the world.

In this study, participants completed a four-item measure of acculturation that reflects language preferences (in speech, writing, thought, and social and vocational contexts); lower scores indicate a lower level of English language acculturation [18]. The participants then completed all items in the PRO-CTCAE-Simplified Chinese item library (124 items for females; 121 items for males). Interviewers did not aid study participants in responding to the PRO-CTCAE-Simplified Chinese items. Participants indicated those PRO-CTCAE items they found difficult to understand or difficult to answer. Interviewers also took field notes to record behavioural indicators of possible non-comprehension such as hesitation with responses, changing of responses, or participants’ facial expressions or body language that might suggest uncertainty and thus lack of comprehension of the symptom items. After the participant completed the PRO-CTCAE items, the interviewer conducted cognitive debriefing interviews in Mandarin Chinese. Verbal probing was used to elicit the participants’ comprehension of the symptom terms (e.g. what does the word ‘fatigue’ mean to you?), and the attributes (e.g. what does the phrase ‘severity at its worst’ mean in this question?). In addition, the probes elicited the participants’ cognitive processes regarding the distinctions among frequency, severity, and inference, and the recall period, as well as their judgment processes in selecting a response choice [15, 19]. Probing also focused on those items where a participant had appeared to be hesitant or uncertain in selecting a response, and items the participant had marked as difficult to understand or difficult to answer. If a participant did not indicate that any items as difficult to understand or difficult to answer, the interviewers probed on PRO-CTCAE items for loose or watery stools, shortness of breath, problems with memory, decreased appetite, insomnia, increased passing of gas, flashing lights in front of your eyes, watery eyes, and wheezing, as these were symptom terms where challenges arose in the translation process. All interviews were scheduled to coincide with participants’ clinical visits; interviews were audio-recorded and conducted in a private room at the study site.

### Analysis

The audio-recorded interviews were transcribed verbatim, and the transcript was verified by another research staff member. Interview data pertaining to symptom terms and attributes were summarized and analysed across participants. Linguistic and cultural themes related to comprehension, cultural appropriateness and relevance, and cognitive processes were categorized, and subsequently analysed to determine semantic and conceptual equivalence with the original English PRO-CTCAE phrasings. In addition, the proportions of participants who experienced comprehension difficulties with respect to the symptom terms, and the attributes (e.g. frequency, severity, interference) were tabulated. Generally, items indicated as difficult to understand or difficult to answer by ≥20% of participants during Round 1 of the cognitive debriefing interviews were reviewed by the study team and considered for revision and retesting in Round 2. Items experienced as difficult by less than 20% of the sample were also reviewed, and if better phrasing options were available, revisions were made and retested in Round 2. All decisions regarding revisions considered the interview data, behavioural indicators of possible non-comprehension from the field notes, the characteristics of the participants (e.g. education level), and the availability of suitable alterative phrasings [4].

## Results

During the period of September 2012–December 2015 (except October 2013–March 2014), 81 participants were enrolled in this study, completed the initial version of the PRO-CTCAE-Simplified Chinese, and participated in the first round of cognitive debriefing interviews. From May to December 2016, 15 additional participants enrolled and participated in a second round of cognitive debriefing to evaluate items that were revised after Round 1. The socio-demographic and clinical characteristics of the interview samples in both rounds were comparable. In the pooled sample, slightly more than 60% of the participants were aged 50–64 years, and more than 80% were female. Approximately one third of participants had stage 1 or II disease: a majority of the sample had received treatment with surgery and chemotherapy. Almost one-third (30.2%) had completed only primary education (approximately equivalent to 6th Grade or less). The sample also had a low level of English acculturation, with more than 80% reporting that they were most comfortable speaking Chinese, and preferred to speak Chinese with friends, to think in Chinese, and to speak Chinese at home (Table 1). The mean duration of the interviews was 15 min with a range of 6 to 36 min.

**Table 1:** Sample characteristics

Characteristics	Pooled sample Rounds 1 & 2 (*N* = 96)	Round 1 (*n* = 81)	Round 2 (*n* = 15)
**Age in years (mean ± SD)**	55.16 ± 8.7	55.05 ± 8.7	55.87 ± 9.8
< 50	23 (24.0)	20 (24.7)	3 (20)
50–64	61 (63.5)	52 (64.2)	9 (60)
≥ 65	12 (12.5)	9 (11.1)	3 (20)
**Gender**			
Female	80 (83.3)	68 (84)	12 (80)
**Education level**			
No formal education	2 (2.1)	2 (2.5)	0
Primary	29 (30.2)	26 (32.1)	3 (20)
Secondary	54 (56.3)	44 (54.3)	10 (66.7)
Junior college/diploma	8 (8.3)	6 (7.4)	2 (13.3)
Undergraduate to postgraduate	3 (3.1)	3 (3.7)	0
**Country of birth**	*(n = 94)*	*(n = 79)*	
China	8 (8.5)	6 (7.6)	2 (13.3)
Hong Kong	1 (1.1)	1 (1.3)	0
Malaysia	16 (17.0)	15 (19)	1 (6.7)
Singapore	69 (73.4)	57 (72.2)	12 (80)
**Language acculturation**			
75–100% feel most comfortable speaking Chinese	79 (82.3)	65 (80.2)	14 (93.3)
75–100% prefer to speak Chinese with friends	77 (80.2)	64 (79)	13 (86.7)
75–100% think in Chinese	82 (85.4)	69 (85.2)	13 (86.7)
75–100% speak Chinese at home	79 (82.3)	67 (82.7)	12 (80)
**Chinese-speaking region or dialect**			
Fujian	39 (40.6)	34 (42.0)	5 (33.3)
Chaoshan	22 (22.9)	17 (21.0)	5 (33.3)
Fujian & Chaoshan	3 (3.1)	3 (3.7)	0
Southern region of Guangdong & Hong Kong	10 (10.4)	8 (9.9)	2 (13.3)
North-eastern region of Guangdong	5 (5.2)	4 (4.9)	1 (6.7)
Hainan	4 (4.2)	3 (3.7)	1 (6.7)
Others	6 (6.3)	5 (6.2)	1 (6.7)
Missing	7 (7.3)	7 (8.6)	0 (0)
**Type of cancer**			
Breast	72 (75.0)	60 (74.1)	12 (80)
Colorectal	24 (25.0)	21 (25.9)	3 (20)
**Stage of cancer**			
I-II	56 (58.3)	48 (59.3)	8 (53.3)
III	34 (35.4)	28 (34.6)	6 (40.0)
issing	6 (6.3)	5 (6.2)	1 (6.7)
**Type of cancer therapy**			*(n = 14)*
Chemotherapy	7 (7.4)	3 (3.7)	4 (28.6)
Surgery + chemotherapy	80 (84.2)	70 (86.4)	10 (71.4)
Surgery + chemo-radiotherapy	8 (8.4)	8 (9.9)	0

Table 2 summarizes the proportions of participants who indicated that an item was difficult to understand or difficult to answer, together with the items that were rephrased after Round 1, and the final decisions about item phrasing that were made based on the data from Round 2. The symptom term ‘Stretch marks’ was considered confusing, hence affecting ease of response by 22.2% of the participants in Round 1. Interview data and field notes revealed that participants were uncertain if the symptom term of ‘Stretch marks’, referred only to stretch marks that related to pregnancy. This symptom term was revised by adding a Chinese word of ‘skin’ before the phrasing of ‘lines on the body’ and was retested in Round 2. There were no further difficulties with this revised item when tested in 15 participants.

**Table 2:** Symptom terms and attributes presenting difficulties with comprehension, clarity or ease of response and the decisions about item phrasing that were made based on the data from two rounds of cognitive debriefing interviews (Round 1, *n* = 81 including 68 females 13 males; Round 2 *n* = 15 including 12 females 3 males)

PRO-CTCAE Symptom Terms					
Source of Difficulty	Proportion with Difficulties in Round 1 (*n* = 81)	PRO-CTCAE Symptom Term % (*f*/n)	Examples of Difficulties Experienced by Participants in Round 1	Decisions based on data from Round 1	Decisions based on data from Round 2
*Comprehension*	≥ 20% with difficulties	None			
	≥ 10% and < 20% with difficulties	Bed sores 17.3% (14/81)	Participants (14/81) did not understand what was meant by ‘bed sore’.	Rephrased to include an elaboration and retested	Well comprehended (15/15) and phrasing retained
		Difficulty getting or keeping an erection 15.4% (2/13 males)	Participants (2/13) did not understand what was meant by ‘erection’.	No suitable alternative term; retested	Well comprehended (3/3 males) and phrasing retained
		Ejaculation problem 15.4% (2/13 males)	Participants (2/13) did not understand what was meant by ‘ejaculation’.	No suitable alternative term; retested	Well comprehended (3/3 males) and phrasing retained
		Increased passing of gas 16% (13/81)	Participants (9/81) did not understand what was meant by ‘passing of gas’.	Rephrased and retested	Well comprehended (15/15) and phrasing retained
	< 10% with difficulties	Abdominal pain 1.2% (1/81)	Participant (1/81) did not understand what was meant by ‘abdominal pain’. Some participants suggested other plain language Chinese phrasing (synonyms) that would be more culturally acceptable to the Chinese-speaking population from southern region.	Included synonyms and retested	Well comprehended (15/15) and phrasing retained
		Bloating 1.2% (1/81)	Participant (1/81) did not understand what was meant by ‘bloating’. Some participants suggested alternate plain Chinese language phrasing (synonyms) that would be more culturally acceptable to the Chinese-speaking population from southern region.	Included synonyms and retested	Well comprehended (15/15) and phrasing retained
		Flashing lights in front of your eyes 1.2% (1/81)	Participant (1/81) did not understand what was meant by ‘flashing lights’. Some participants’ (3/81) facial expressions suggested uncertainty about the intended meaning of this symptom term.	Rephrased and retested	Well comprehended (15/15) and phrasing retained
		Frequent urination 6.2% (5/81)	Participants (4/81) did not understand what was meant by ‘frequent urination’. Some participants suggested alternate simple Chinese language phrases (synonyms) that would be more culturally acceptable to the Chinese-speaking population from southern region.	Included synonyms and retested	Well comprehended (15/15) and phrasing retained
		Numbness or tingling in your hands or feet 1.2% (1/81)	Participant (1/81) did not understand what was meant by ‘numbness or tingling’. Some participants’ (3/81) facial expressions suggested uncertainty about the intended meaning of this symptom term.	Rephrased and retested	Well comprehended (15/15) and phrasing retained
		Wheezing 2.5% (2/81)	Participant (1/81) did not understand what was meant by ‘wheezing’. Participants recommended an elaboration to improve comprehension and clarity.	Rephrased, included an elaboration, and retested	Well comprehended (15/15) and phrasing retained
*Ease of recall, judgment and response*	≥ 20% with difficulties	Stretch marks 22.2% (18/81)	Participants (18/81) were uncertain if it related to pregnancy.	Rephrased and retested	Well comprehended (15/15) and phrasing retained
	≥ 10% and < 20% with difficulties	None			
	< 10% with difficulties	Hot flashes 6.2% (5/81)	Participants (3/81) were uncertain if it related to fever or feeling hot. Some participants recommended an elaboration to improve comprehension and clarity.	Item rephrased to include an elaboration and retested	Well comprehended (15/15) and phrasing retained
		General pain 4.9% (4/81)	Participants (3/81) were uncertain about the type/ location of pain. Some participants recommended an elaboration to improve comprehension and clarity.	Item rephrased to include an elaboration and retested	Well comprehended (15/15) and phrasing retained
		Nausea 4.9% (4/81)	Participants (4/81) confused this terminology as ‘vomiting’. Some participants suggested to include some elaborations.	Rephrased, added an elaboration, and retested	Well comprehended (15/15) and phrasing retained
		Vaginal dryness 4.4.% (3/68 females)	Participants (2/68) were uncertain about the context of condition. Some participants (5/68) showed hesitation with responses	Rephrased and retested	Well comprehended (12/12 females) and phrasing retained
		Nothing could cheer you up 3.7% (3/81)	Participants (3/81) had difficulty choosing a response. Some participants (2/81) showed hesitation with responses	Rephrased and retested	Well comprehended (15/15) and phrasing retained
		Pain during vaginal sex 2.9% (2/68 females)	Participants (2/68) had difficulty choosing a response. Some participants (5/68) showed hesitation with responses	Rephrased and retested	Well comprehended (12/12) and phrasing retained
		Pounding or racing heartbeat 2.5% (2/81)	Participants (2/81) did not understand to what extend to be considered as pounding. Some participants (2/81) showed hesitation with responses	Rephrased and retested	Well comprehended (15/15) and phrasing retained
		Sad or unhappy feelings 1.2% (1/81)	Participant (1/81) had difficulty choosing a response. Some participants (3/81) showed hesitation with responses.	Rephrased and retested	Well comprehended (15/15) and phrasing retained
		Urinary urgency 1.2% (1/81)	Participant (1/81) did not understand to what extend to be considered as urge. Some participants suggested alternate simple Chinese language phrases (synonyms) that would be more culturally acceptable to the Chinese-speaking population from southern region.	Included synonyms and retested	Well comprehended (15/15) and phrasing retained
PRO-CTCAE Attributes					
Source of Difficulty	Proportion with difficulties in Round 1 (*n* = 81)	PRO-CTCAE Attribute % (n/N)	Examples of Difficulties Experienced by Participants in Round 1	Decisions based on data from Round 1	Decisions based on data from Round 2
*Comprehension*	≥ 20% with difficulties	None			
	≥ 10% and < 20% with difficulties	None			
	< 10% with difficulties	Interfere 8.6% (7/81)	Participants (4/81) did not understand what was meant by ‘interfere’.	No suitable alternative term; retested	Well comprehended (15/15) and phrasing retained
*Ease of recall, judgement and response*	≥ 20% with difficulties	Severity at its Worst 23.5% (19/81)	Participants (11/81) commented this was a double barrelled phrasing, making it difficult to choose a response.	Rephrased and retested	Well comprehended (15/15) and phrasing retained
	≥ 10% and < 20% with difficulties	How Often 11.1% (9/81)	Participants (9/81) were unclear if this question was asking for them to provide precise count of the number of times something occurred, thus making it difficult to select from the response choices.	Rephrased and retested	Well comprehended (15/15) and phrasing retained
	< 10% with difficulties	None			

PRO-CTCAE-Simplified Chinese items reflecting four PRO-CTCAE symptom terms (bed sores, difficult getting or keeping an erection, ejaculation problems, increased passing of gas) were identified as difficult to comprehend by 10–20% of respondents. For ‘Bed sores’ and ‘Increased passing of gas’, alternative phrasings were proposed by the study team and these items were revised and retested with 15 participants in Round 2. There were no further difficulties with these revised symptom terms in Round 2 of the cognitive debriefing interviews. Since plain language alternative phrasings for ‘Difficulty getting or keeping an erection’ and ‘Ejaculation problem’ were not available, they remained unchanged. None of the 15 participants in Round 2 endorsed comprehension difficulties with these symptom terms.

Fewer than 10% of respondents found that symptom terms addressing flashing lights in front of your eyes, numbness or tingling in your hands or feet, wheezing, hot flashes, general pain, nausea, vaginal dryness, sad or unhappy feelings, feeling that nothing could cheer you up, pain during vaginal sex, pounding or racing heartbeat, bloating, abdominal pain, frequent urination, and urinary urgency were difficult to comprehend or difficult to answer. As described below and in Table 2, alternative phrasings were proposed by either interviewers and/or respondents, and items were revised and retested with 15 participants in Round 2. There were no further difficulties with those revised symptom terms in Round 2 of the cognitive debriefing interviews.

For example, interview data and field notes from Round 1 of the cognitive debriefing interviews revealed that some participants were uncertain about the symptom terms of ‘Hot flashes’ (e.g. feeling hot, fever, etc.) and the type/ location of general pain (e.g. head, back, etc.), and recommended that an elaboration be included in parentheses. Therefore, elaborations (feeling of intense heat that may be accompanied with sweating and rapid heartbeat for ‘Hot flashes’; experienced in any parts of the body for ‘General pain’) were added to these two symptom terms, resulting in improved clarity and comprehension. A few participants in Round 1 of the cognitive debriefing interviews suggested synonyms for abdominal pain, bloating, frequent urination, and urinary urgency that they believed would be more culturally acceptable and better comprehended by Chinese-speaking respondents from the southern region. Therefore, the study team decided to incorporate synonyms into items to strengthen the comprehension and cultural acceptability of these items for a diverse range of Chinese speakers. The 15 participants in Round 2, including two from the southern region of Guangdong, indicated that the inclusion of these synonyms improve comprehension and clarity.

Data from Round 1 revealed that the phrasing of the PRO-CTCAE attributes for severity, frequency, and interference was considered difficult to comprehend or difficult to judge by 19 (23.4%), nine (11.1%), and seven (8.6%) out of 81 participants, respectively. Some participants commented that the expression of ‘severity at its worst’ in Chinese seemed to contain a double-negative, and that as a result it was difficult to select a response option. This element of the PRO-CTCAE translation was reviewed by the study team and was revised to avoid this unintended double-negative phrasing, and to improve the comprehension and clarity of ‘severity at its worst’ for Chinese speakers. There were no further difficulties when the revised phrasing of ‘severity at its worst’ was retested in Round 2 with 15 participants. Participants also commented that the phrasing of the frequency question ‘how often’ was interpreted as requesting a specific count of the number of times something occurred in the past 7 days. As such, respondents felt that the response choices offered (never, occasionally, sometimes, etc.) did not seem well matched to the question phrasing, making it difficult for them to select a response. Better alternative phrasing was available for these frequency items, and there were no further difficulties when the revised phrasing was retested with 15 participants in Round 2. Since better alternative phrasing was not available for the attribute of ‘interfere’, it remained unchanged.

No patterns in reported comprehension difficulties were observed in subgroups based on educational level, gender or Chinese-speaking geographical region. No participant in Round 1 or Round 2 reported difficulty with the phrasings chosen for the 7-day recall period or the response options. Furthermore, none of the 15 participants in Round 2 reported difficulties in understanding any of the symptom terms including the modified symptom terms and attributes, and thus acceptable comprehension of the finalised version of the PRO-CTCAE-Simplified Chinese was confirmed.

## Discussion

Sireci (1998) has argued that demonstration of content validity is a fundamental requirement of all assessment instruments [20]. In this study, a cognitive debriefing interview approach [14–16] to content validation provides evidence that the finalized PRO-CTCAE-Simplified Chinese was clearly understood by patients with breast or colorectal cancer from diverse Chinese-speaking regions and with various levels of educational attainment. Our translation and linguistic validation team was comprised of Chinese health care professionals from Singapore, China, and Hong Kong SAR, China who were well-versed in spoken/ written Chinese and English, thereby ensuring that PRO-CTCAE-Simplified Chinese items were culturally relevant and acceptable for use across different Chinese-speaking populations. Our approach to translation and linguistic validation including independent forward and back translations, reconciliation and harmonization, and iterative cognitive debriefing interviews to strengthen and establish conceptual equivalence with PRO-CTCAE English source. Indeed, the inclusion of cognitive debriefing interviews in the process of linguistic validation is crucial to ensure cross-cultural content validity of a translated and adapted patient-reported outcome measure [21].

Caveats when interpreting the results of our study include that our sample was comprised of patients with breast or colorectal cancer, and that there were only 16 male participants (13 interviews in Round 1, and 3 interviews in Round 2) in the pooled sample. At the same time, our large sample size and enrichment for lower educational attainment and lower English language acculturations strengthens confidence in the generalizability of our results. Literature suggests that cognitive interviewing studies require approximately 5–15 interviews per item [14].

With our large sample size, we were able to ensure that at least 13 participants or more were debriefed about each of the 124 PRO-CTCAE items. Although among our 13 male respondents in Round 1, there were a few difficulties with comprehension of male-specific symptom terms, there were no other observed patterns by gender with respect to the comprehension, clarity or ease of response of the gender non-specific PRO-CTCAE symptom terms.

Of note, we found it challenging to develop item phrasings for the PRO-CTCAE question stems reflecting the attributes of frequency and ‘severity at its worst’. As the English and Chinese language differ considerably in terms of the grammar and syntax for phrasing questions, phrasing with respect to frequency and severity in the initial PRO-CTCAE-Simplified Chinese version aimed to achieve conceptual equivalence with the English PRO-CTCAE. Thus, during forward translation, the translators employed phrasing that corresponded closely to the English language version. The back translation also reflected conceptual equivalence to the English PRO-CTCAE source material. Nevertheless, 23.4 and 11.1% of the participants in Round 1 found the chosen phrasings for the PRO-CTCAE attributes ‘severity at its worst’ and ‘how often’, respectively, challenging to interpret in light of the response options. Specifically, the participants understood the expression of ‘severity at its worst’ in Chinese as a double-negative and interpreted questions asking ‘how often’ as requesting a specific count of the number of times they experienced a symptom. The study team subsequently revised the Chinese language phrasings of these attributes to achieve improved comprehension and ease of response by Chinese-speaking participants, while also maintaining conceptual equivalence with the English PRO-CTCAE. No further difficulties were reported when the revised attributes of ‘severity at its worst’ and ‘how often’ were retested with all of the 15 participants in Round 2.

In this study, phrasings used for PRO-CTCAE-Simplified Chinese symptom terms were generally well-understood, and meaningful to diverse respondents, Overall, our data did not reveal any distinct patterns of difficulty by geographical region or level of educational attainment. At the same time, the interpretation by Chinese speakers of the phrasing chosen for a few specific PRO-CTCAE terms (e.g. abdominal pain, bloating, frequent urination, and urinary urgency) did vary based on geographic region. Accordingly, we added synonyms alongside the original translated phrasings to enhance the culturally acceptability of these symptom terms for diverse Chinese-speaking populations. The participants in Round 2 appreciated the addition of these synonyms, and indicated that their inclusion strengthened comprehension and clarity. The cognitive debriefing interviews conducted in Rounds 1 and 2 further suggested that the recall period, response options, and general instructions were understandable and comprehensible.

Our study results should be interpreted in light of a few limitations. First, our sample may have been biased toward inclusion of participants with preserved performance status since completion of the PRO-CTCAE-Simplified Chinese survey and the cognitive debriefing interview required that participants have the necessary stamina to complete these study-related procedures. Second, all study participants were residents of Singapore. While we believe that the clinical, demographic, and geographical diversity of our sample support our conclusions about content validity, additional testing to confirm the comprehensibility and cultural acceptability of PRO-CTCAE-Simplified Chinese in other countries is warranted. Third, while our sample was quite large and diverse with respect to geographic region, age and educational attainment, we only sampled patients with breast and colorectal cancer, and the sample was predominantly female. Although we did not observe differences between men and women in the comprehension of gender non-specific symptom terms, the underrepresentation of males in our sample limits our ability to conclude that PRO-CTCAE-Simplified Chinese is well-comprehended by male respondents. Future studies should evaluate PRO-CTCAE-Simplified Chinese with respondents being treated for a broader range of tumor types, and in samples with more balanced gender distribution. At the same time, confidence in the generalizability of our study findings is strengthened by the rigorous process of translation and cognitive interviewing, and the large sample of geographically diverse group of respondents with low English acculturation.

## Conclusion

Our results support the content validity and acceptability of PRO-CTCAE-Simplified Chinese, and suggest that this translation can be used to capture symptomatic toxicities of cancer treatment in Chinese speakers. Replication and extension of these findings with Chinese speakers residing in other countries is ongoing, and will provide additional evidence to support the comprehensibility and cultural acceptability of PRO-CTCAE-Simplified Chinese in male respondents and for those respondents being treated for a broader range of tumor types. Additional studies to quantitatively examine the reliability and construct validity of PRO-CTCAE-Simplified Chinese are also warranted.

Going forward, PRO-CTCAE-Simplified Chinese provides a common platform by which trialists can capture treatment-related side effects by patient self-report from Chinese speakers. Such information can be used to identify effective and tolerable cancer treatment regimens, to improve shared decision-making between clinicians and patients, and to guide provision of targeted supportive care to improve tolerability.

## Data Availability

The data and materials are available from the corresponding author on reasonable request.

## Abbreviations

CTCAE: Common Terminology Criteria for Adverse Events
NCI: National Cancer Institute
PROs: Patient-reported outcomes
PRO-CTCAE: Patient-Reported Outcomes version of the Common Terminology Criteria for Adverse Events
